# Barriers to evidence-based physiotherapy practice for stroke survivors in Ghana

**DOI:** 10.4102/sajp.v74i1.423

**Published:** 2018-05-31

**Authors:** Jonathan Quartey, Samuel Kwakye

**Affiliations:** 1 Department of Physiotherapy, University of Ghana, Ghana; 2 Department of Physiotherapy, Police Hospital, Cantonments, Accra, Ghana

## Abstract

**Background:**

Physiotherapy has evolved over the years, and this has led to an increasing demand in using evidence as a basis for making clinical decisions because evidence-based interventions for stroke have been shown to be effective. However, the inability to carry out any of the evidence-based practice (EBP) processes may constitute a barrier to its application in practice.

**Aim:**

To determine the barriers to EBP of physiotherapy services for stroke survivors in Ghana.

**Methods:**

A cross-sectional study that involved 121 physiotherapists of the Ghana Physiotherapy Association providing services to stroke survivors. Physiotherapists completed a self-administered questionnaire. Logistic regressions were used to examine relationships between socio-demographic and practice characteristics of respondents and each practitioner factor. A logistic regression was used to identify the association between organisational characteristics and each organisational factor that facilitates EBP.

**Results:**

Self-efficacy ratings for performing EBP were below 50% for critical appraisal of the literature and interpretation of statistics. All the participants stated that they had organisational challenges, which tend to affect the implementation of evidence-based physiotherapy practice for stroke. The five most reported barriers to updating knowledge on EBP included lack of organisational mandate (56.2%), insufficient time (46.3%), lack of information resources (43%), lack of understanding of statistics (35.5%) and lack of interest (33.1%).

**Conclusion:**

Lack of adequate resources, lack of organisational support and low self-efficacy to perform EBP activities constitute barriers to implementing EBP for stroke survivors.

**Clinical implications:**

Findings of the study reinforce the need to develop a supportive organisational infrastructure to increase research integration in physiotherapy practice.

## Introduction

Evidence-based practice (EBP) is a five-step process through which clinicians integrate research evidence with clinical expertise and patient preferences, producing the most appropriate and effective service (Bello 2011). It includes expressing questions that arise from clinical practice in a searchable format; effectively finding the best evidence to address the question, a step that may require an online literature search; and critically appraising the evidence for validity, impact and applicability to the clinical question (Silva, Costa and Costa 2015). The inability to carry out any of these functions may constitute a barrier to the application of EBP in practice. The Sicily statement in 2005 recommended that every individual practitioner be trained in the five-step model of EBP with skills to ask a research question, access, appraise and apply the evidence, and assess the process (Dawes et al. 2005). However, in Ghana, this has not been categorically embedded in the curriculum of the entry-level education (bachelor’s degree). Hence, physiotherapy undergraduate students access EBP training on their own, either via journals or via online access. A systematic review of studies conducted over the world, describing current evidence of EBP by physiotherapists (PTs), showed that the most frequent barriers reported were lack of time, inability to understand statistics, lack of support from employer, lack of resources, lack of interest and lack of generalisation of results (Mota da Silva, Costa & Garcia 2015). Engaging with both research and clinical findings can enhance the proficiency of PTs’ clinical practice (Bello 2011) and help prevent the misuse, overuse and underuse of health care service (Kumar, Grimmer-Sommers & Hughes 2010).

Over the years, several studies have shown that EBP improves the effectiveness of health service delivery and, consequently, client outcomes. Evidence supporting this finding for stroke has been reported (Langhorne, Bernhardht & Kwakkel 2011). However, even when an intervention has good evidence of benefit, the application of EBP for stroke can still raise challenges (Burt, Lohn & Cohe 2009). For example, a systematic review by Scurlock-Evans and colleagues comprising 32 studies from Europe, Africa and Asia showed that the main barriers faced by PTs were usually related to lack of time and skills, and also misperceptions of EBP. Other considerable barriers related to the workplace include no access to scientific literature at work, no policies at work to stimulate the use of evidence, lack of training at work to use the evidence (Gorgon et al. 2013) (study in Philippines) and an inability to make changes in the workplace (Iles & Davidson 2006) (study in Australia).

Manns, Norton and Darrah (2015) show that the content of EBP, knowledge and skills in physiotherapy have been improved over the years, both in education curricula and in clinical practice, but research evidence is used minimally, and thus the gap between knowledge and practice continues. The assumption was that this knowledge set would result in the increased use of EBP behaviours by graduates. However, both the medical (Flores-Mateo & Agimon 2007) and rehabilitation (McEvoy et al. 2011) literature conducted in Australia suggest that a knowledge-to-practice gap exists, with a lack of uptake of EBP skills into clinical decision-making. Studies on barriers to EBP have been carried out among PTs in the United States (Jette et al. 2003), United Kingdom (Pollock et al. 2014) and Canada (Salbach et al. 2007). Similar barriers to EBP were reported in these countries, which included lack of time, misperceptions of EBP and lack of understanding of statistical analysis. However, differences in entry-level training, health care systems and professional practice across countries limit the generalisability of those studies to the Ghanaian environment.

There is evidence to support stroke rehabilitation in well-coordinated multidisciplinary stroke units or through provisions of early supported provision of discharge teams (Langhorne et al. 2011). Potentially, beneficial treatment options for motor recovery of the arm include constraint-induced movement therapy (CIMT) and robotics (Langhorne et al. 2011). In addition, the integration of electrical stimulation and physiotherapy is feasible and tends to improve gait post-stroke (Wilkinson et al. 2015). Teasell et al. (2015) in a study of evidence-based stroke rehabilitation showed that there is a high level of evidence for stroke rehabilitation. However, by anecdotal observations during clinical rotations, the use of research findings in clinical practice is not very common among PTs in Ghana. There also seems to be a dearth of information on barriers to evidence-based physiotherapy (EBPT) practice in Africa. Thus, the primary objective of this study was to determine the barriers to the implementation of EBP by PTs for stroke survivors in Ghana. A secondary objective was to identify associations between organisational characteristics and each organisational factor to facilitate EBP.

## Methods

A cross-sectional survey of PTs who are providing or have provided services to people with stroke in Ghana was conducted. All 130 members of the Ghana Physiotherapy Association (GPA) were recruited for the study using a convenience sampling technique. The study was conducted in both private and public physiotherapy facilities in Ghana. PTs were considered eligible for the study if they had two or more years of working experience and were registered members of GPA. Physiotherapists who were not registered members of GPA, had less than 2 years working experience and had not treated people with stroke before were ineligible for the study.

### Data collection tool

The ‘barriers to evidence-based physical therapy practice for people with stroke’ questionnaire used by Salbach et al. (2007) was the instrument adapted for this study. The instrument was a one-time questionnaire that included 40 items of which the majority required participants to indicate their level of agreement with a statement on a 5-point Likert scale. Those recipients who indicated in the first item that they did not provide services to people with stroke were excluded and were asked to leave the rest of the questionnaire blank. Questionnaire items (Appendix 1) were designed to identify practitioner and organisational factors influencing EBP. Subgroups of items were used to evaluate education about EBP (items 13–15), attitudes and beliefs (items 2, 3, 5 and 7), interest (items 4 and 6) and perceived role (items 11 and 12) to engage in EBP, self-efficacy to perform EBP activities (items 16.1–16.12), perceived organisational and peer support for EBP (items 23 and 24), and organisational resources and support to promote EBP (items 17–22). One item was used to identify the three greatest barriers to updating clinical practice with new knowledge (item 25). Items were added to the end of the questionnaire to evaluate respondent demographics, practice characteristics and work setting (items 26–40). The questionnaire was developed by pooling the primary source of items from a survey tool used by Jette et al. (2003) to evaluate PTs’ beliefs, attitudes, knowledge and behaviour in relation to EBP. Items were added to assess EBP beliefs and the existence of an organisational mandate supporting EBP. Three new items to evaluate physical therapists’ perceived role in searching and appraising the research literature and interpreting its applicability to individual clients were devised based on investigations by other researchers.

Self-efficacy to perform EBP activities (items 16.1–16.12) was measured using a 12-item scale that was developed in adherence to guidelines for developing self-efficacy scales. Participants were asked to rate their level of confidence in their ability to perform each activity, using an 11-point scale ranging from 0% (‘cannot do at all’) to 100% (‘certain can do’).

### Procedure

Potential participants were asked to indicate in the first item of the questionnaire whether they provide or have provided services to stroke survivors or not. Follow-ups were made via phone calls, e-mails and text messages twice every week to serve as reminders because most participants complained of busy schedules at their workplaces. Participants in other regions were required to submit the questionnaire within 4 weeks to a research assistant who in turn returned them personally or by post to the first author. Participants who received the questionnaire by e-mail submitted via the same channel and the questionnaires were then printed out. Copies of questionnaires were retrieved personally from some participants. Of the 130 registered PTs contacted, 121 responded, representing a 93% response rate.

The data were analysed using the Statistical Package for Social Scientists (SPSS), version 23, to run basic descriptive statistics such as frequencies, percentages and means of the main variables of investigation. The prevalence of practitioner and organisational factors was estimated using percentages. A logistic regression was used to examine relationships between socio-demographic and practice characteristics of respondents (i.e. independent variables) and each practitioner factor (i.e. dependent variable). Independent variables included age, sex, highest degree obtained, number of years practising, number of hours worked per week, number of patients seen per day, care delivery within a multidisciplinary team (MDT) and supervision of physiotherapist students. Dependent variables were items used to determine education about EBP, attitudes and beliefs, interest and perceived role in EBP, and self-efficacy to perform EBP activities.

A logistic regression was also used to identify associations between organisational characteristics (independent variables), including facility location and type, number of physical therapists at the facility, and status as a teaching institution, and each organisational factor (dependent variable), including items measuring perceived peer and organisational support and the existence of resources (e.g. access to journals, Internet and personnel) to facilitate EBP.

In order to use a logistic regression, categories were combined to produce binary-dependent variables. For instance, for statements with a positive response set using a Likert scale, the ‘strongly agree’ and ‘agree’ categories were collapsed to form an ‘agree’ category, and the ‘neutral’, ‘disagree’ and ‘strongly disagree’ categories were combined to form a ‘disagree’ category. For items with a negative response set, the ‘strongly disagree’ and ‘disagree’ categories were collapsed to form a ‘disagree’ category, and the ‘neutral’, ‘agree’ and ‘strongly agree’ categories were combined to form an ‘agree’ category. Categories of demographic variables with low cell counts also were collapsed in order to obtain stable estimates in the regression analyses. A logistic regression was then performed to estimate the influence of each independent variable on a dependent variable. Odds ratios (ORs) and associated 95% confidence intervals (CIs) were reported for statistically significant associations. The alpha level was set at *p* = 0.05.

### Ethical consideration

Ethical approval (SBAHS/10403342/AA/R4/2015-2016) was obtained from the Ethics and Protocol Review Committee of the School of Biomedical and Allied Health Sciences, University of Ghana.

## Results

The final sample consisted of 74 males (61.2%) and 47 females (38.8%) between 20 and 40 years of age (mean = 30 ±6). A bachelor’s degree (91.7%) was the most commonly cited highest degree obtained and 68 (56.2%) of the respondents reported having between 5 and 8 years of practice experience. Table 1 shows respondent and practice characteristics.

**TABLE 1:** Characteristics of participants and their practice (*N* = 121).

Characteristics	*N*	%
**Age (years)**		
20–25	16	13.2
26–30	63	52.1
31–35	29	23.9
36–40	13	10.7
**Sex**		
Male	74	61.2
Female	47	38.8
**Entry level degree**		
Certificate	121	100.0
**Highest degree**		
Bachelor’s	111	91.7
Entry level master’s	4	3.3
Applied or research master’s	6	5.0
**Years of practice**		
< 5	45	37.1
5–8	68	56.2
> 8	8	6.6
**Hours of work per week**		
< 20	11	9.1
20–30	25	20.7
31–40	81	66.9
> 40	4	3.3
**Number of patients seen per day**		
< 5	-	-
5–10	40	33.1
11–15	39	32.2
> 15	42	34.7
**Number of stroke patients seen in a day**		
< 3	4	3.3
3–5	39	32.2
6–10	38	31.4
> 10	40	33.1
**Clinical instructor**		
Yes	108	89.3
No	13	10.7
**Member of MDT**		
Yes	111	91.7
No	10	8.3

MDT, multidisciplinary team; *N*, number.

Table 2 shows characteristics of the organisations for which the participants worked. The most frequently reported characteristics were an urban location among 107 (88.4%) of the participants. One hundred and three (88.5%) participants worked in a general hospital and 70 (57.9%) participants practised in a teaching hospital. Table 3 shows participants’ educational background, attitudes and beliefs towards EBP and their interests and perceived roles in EBPT. Mostly, 118 (97.5%) participants generally held positive attitudes and beliefs about EBP. Eighty (66.1%) participants disagreed that the adoption of EBP creates unreasonable demands on PTs and 84 (69.4%) participants disagreed that EBP does not take into account patient preferences. The participants were diverse in expressing whether or not they had the knowledge and skills necessary for EBP or in search engines. More than half of the participants, 82 (67.8%), had learned the foundations for EBP as part of their academic preparation and about 81 (66.9%) agreed that they had received formal training in search engines in finding research relevant to their practice.

**TABLE 2:** Characteristics of the practice setting.

Characteristics	*N*	%
**Location of facility**		
Rural	3	2.5
Urban	107	88.4
Suburban	11	9.1
**Type of facility**		
General hospital	103	85.1
Rehab hospital	1	0.8
Community care access centre	1	0.8
Private practice	5	4.1
University or educational institute	11	9.1
**No. of physiotherapists at facility**		
< 5	62	51.2
5–10	21	17.3
11–15	1	0.8
> 15	37	30.5
**Teaching institute**		
Yes	70	57.9
No	51	42.1

**TABLE 3:** Education, attitudes and beliefs, and interest and perceived role in evidence-based practice.

Item	*N*	Response (%)		
		Disagree	Neutral	Agree
**Education, knowledge and skills**				
I learned the foundations for EBP as part of my academic preparation	121	14.0	18.2	67.7
I received formal training in search strategies for finding research relevant to my practice	121	33.0	14.0	52.9
I received formal training in how to critically evaluate research literature as part of my academic preparation	121	52.9	17.4	29.7
**Attitudes or beliefs**				
Application of EBP is necessary in the practice of physical therapy	121	0.8	1.7	97.5
Literature and research findings are useful in my day-to-day practice	121	0.0	7.4	92.6
The adoption of EBP places an unreasonable demand on physical therapists	121	66.1	9.1	24.8
EBP improves the quality of patient care	121	2.5	8.3	89.2
EBP helps me make decisions about patient care	121	9.1	9.1	81.8
EBP does not take into account patient preferences	121	69.4	16.5	14.0
**Interest or perceived role**				
I need to increase the use of evidence in my daily practice	121	0.0	1.7	98.3
I am interested in learning or improving the skills necessary to incorporate EBP into my practice	121	8.3	6.6	85.1
Physical therapists should be responsible for conducting their own literature reviews to answer their clinical questions	121	24.8	22.3	52.9
Physical therapists should be responsible for critically evaluating the quality of the literature to address their clinical questions	121	21.5	9.1	69.4
Physical therapists should be responsible for interpreting whether research findings apply to their individual patients	121	16.5	10.7	72.7

EBP, evidence-based practice; *N*, number.

**FIGURE 1:**
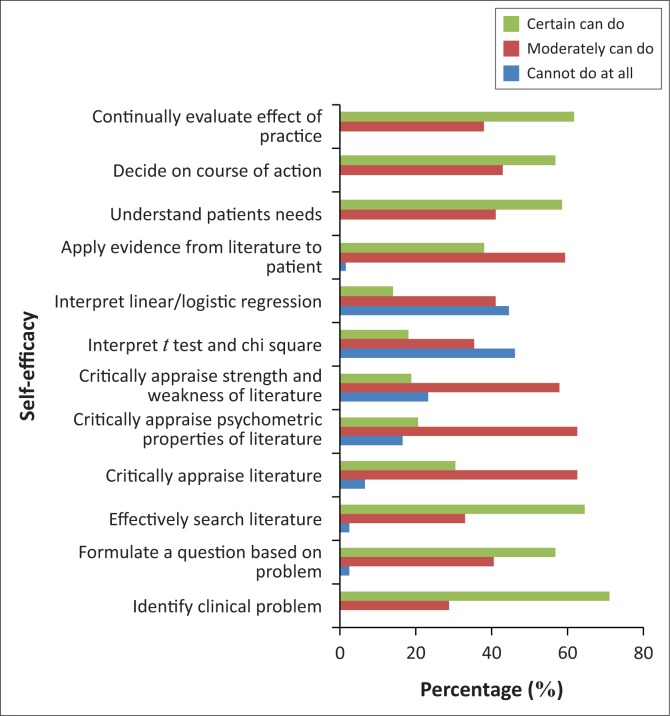
Self-efficacy in performing evidence-based practice for stroke survivors.

Average self-efficacy ratings were between 50% and 70%, for critically appraising the literature for reliability and relevance, the psychometric properties of outcome measures and the strengths and weaknesses of different study designs. Average ratings below 50% were observed for participants’ confidence about their ability to interpret results of statistical procedures such as t-tests, chi-square tests and linear or logistic regression. Self-efficacy ratings for 12 different activities necessary to implement EBP are shown in Figure 1.

**FIGURE 2:**
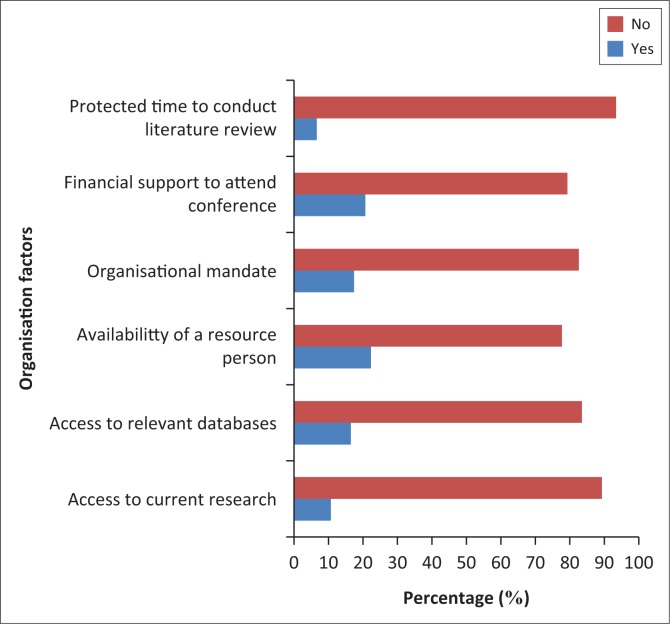
Organisational factors affecting the implementation of EBPT for stroke survivors.

Figure 2 shows the distribution of response related to organisational factors affecting the implementation of EBPT for stroke survivors. All the participants stated that they generally had organisational challenges, which in turn affected the implementation of EBPT practice for stroke. Most (113; 93.4%) of the participants stated that their facilities did not provide protected time to conduct literature reviews and appraise the literature, and 108 (89.3%) of the participants did not have access to current research. One hundred (82.6%) participants stated that their facilities do not mandate the use of current research findings in practice.

It was noticed that age, highest degree attained and number of years practised by participants were associated with academic preparation in EBP and formal training with critical appraisal. Sex was also associated with training in critical appraisal skills with male participants 3.0 times more likely than female participants to report training (95% CI = 0.5–2.1). Age, years practised, hours of work per week and practice in a multidisciplinary team were each associated with formal training with search strategies. For example, compared with participants who had more than 8 years of practice experience, participants with less than 5 years of experience were 18.3 times more likely to have learned the foundations of EBP in their academic preparation (95% CI = 1.8–2.9) and 8.5 times more likely to report having received formal training in critical appraisal skills (95% CI = 5.6–132.7). The relationships between socio-demographic and practice characteristics and EBPT practice are presented in Table 4.

**TABLE 4:** Relationships between socio-demographic and practice characteristics and evidence-based physiotherapy practice.

Factor	Characteristics	Number of participants	Level	Odds ratio (OR)	95% Confidence interval (CI)
Learned foundations in academic programme	Age (years)	121	20–25	Reference	-
			26–30	1.8	0.5–6.1
			31–35	3.9	0.8–19.2
			36–40	6.6	0.8–54.5
	Highest degree		Bachelor’s	Reference	-
			Entry level master’s	0.3	2.3–5.0
			Applied or research master’s	2.5	0.3–24.8
	Years practised		< 5	18.3	3.8–21.9
			5–8	0.2	0.2–1.4
			> 8	Reference	-
Formal training with search strategies	Age (years)	121	20–25	Reference	-
			26–30	1.5	0.2–11.6
			31–35	1.7	0.3–9.4
			36–40	1.9	1.9–0.4
	Years practised		< 5	8.5	2.0–3.6
			5–8	0.9	0.1–4.3
			> 8	Reference	-
	Hours of work per week		20	0.2	0.1-1.3
			20–30	0.3	0.1–5.2
			31–40	1.2	0.1–17.0
			> 40	Reference	-
	Member of multidisciplinary team		No	Reference	-
			Yes	1.9	0.4–8.6
Formal training with critical appraisal	Age (years)	121	20–25	2.1	0.9–5.0
			26–30	0.8	1.0–4.4
			31–35	0.3	1.0–2.9
			36–40	Reference	-
	Sex		Female	Reference	-
			Male	3.0	0.5–2.1
	Highest degree		Bachelor’s	Reference	-
			Entry level master’s	6.5	0.5–1.4
			Applied master’s	21.4	0.1–1.0
	Years practiced		< 5	40.5	5.6–132.7
			5–10	10.5	3.2–26.3
			11–15	7.5	3.7–15.5
			> 15	Reference	-

Location and type of facility, the number of full-time PTs and status as a teaching institution were associated with perceived organisational resources to support EBP. Compared with urban settings, organisations in rural settings were likely to provide journals in paper format, Internet access or a resource person to assist EBP. Physiotherapists working in a teaching institution were 5.0 times more likely than participants working in a non-teaching institution to report receiving financial support. Table 5 shows the relationship between organisational characteristics and each organisational factor.

**TABLE 5:** Relationship between organisational characteristics and organisational factors and evidence-based physiotherapy practice.

Factor	Characteristics	Level	Odds ratio (OR)	95% Confidence Interval (CI)
Facility provides journals in paper format	Location of setting	Rural	0.02	0.2–0.7
		Urban	Reference	-
		Suburban	3.5	0.2–1.7
	Type of setting	General hospital	21.88	0.3–0.7
		Rehab hospital	0.62	0.3–0.8
		Community care centre	0.05	0.5–0.7
		Private practice	0.76	1.5–7.5
		University or educational institute	1.34	0.2–1.4
	Number of physiotherapists	< 5	0.4	1.1–1.8
		5–10	1.0	1.2–2.3
		11–15	1.5	0.4–1.1
		> 15	1.7	1.7–2.3
	Teaching institution	Yes	3.8	1.5–7.4
		No	2.3	1.0–2.4
Facility provides Internet access	Location of facility	Rural	0.5	2.0–3.4
		Urban	Reference	-
		Suburban	1.8	0.4–0.7
	Type of setting	General hospital	4.8	0.7–7.0
		Rehab hospital	4.3	0.4–2.1
		Community care centre	1.4	1.3–4.0
		Private practice	5.1	2.3–7.4
		University or educational institute	4.9	0.4–6.0
	Number of physiotherapists	< 5	5.1	1.3–4.0
		5–10	6.4	0.4–0.7
		11–15	8.0	1.6–2.5
		> 15	7.0	0.6–3.0
Facility has a resource person to assist with evidence-based practice (EBP)	Location of setting	Rural	0.2	0.5–0.9
		Urban	Reference	-
		Suburban	0.4	0.2–6.6
	Type of setting	General hospital	2.1	1.5–2.0
		Rehab hospital	3.4	0.6–6.1
		Community care centre	0.3	05–1.4
		Private practice	0.2	0.5–5.6
		University or educational institute	4.0	1.4–15.3
	Number of physiotherapists	< 5	Reference	-
		5–10	5.0	2.4–5.7
		11–15	10.2	0.5–1.6
		> 15	11.4	3.4–7.7
	Teaching institute	Yes	4.2	0.6–2.9
		No	2.4	1.8–5.6
Facility mandates the use of research in practice	Number of physiotherapists	< 5	References	-
		5–10	0.3	0.9–2.1
		11–15	1.2	0.3–3.5
		> 15	2.2	0.7–4.0
Facility provides financial support for continuing education	Type of setting	General hospital	Reference	-
		Rehab hospital	1.23	0.2–0.9
		Community care centre	0.1	0.1–0.2
		Private practice	0.2	0.1–4.3
		University or educational institute	1.0	0.2–4.3
	Facility is a teaching hospital	Yes	5.0	1.5–16.7
		No	Reference	-

**FIGURE 3:**
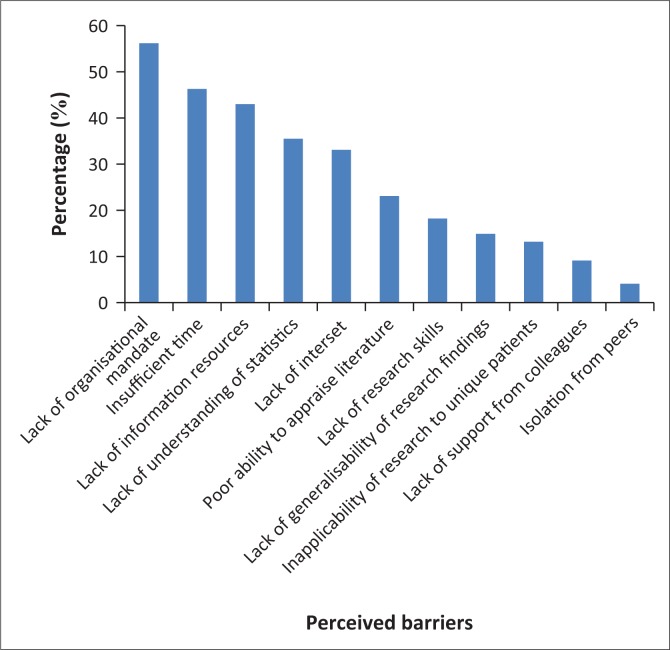
Perceived barriers to evidence-based physiotherapy practice.

Figure 3 shows perceived barriers to updating clinical practice with new information noted by more than 10% of the participants. The most frequent reported barrier was a lack of organisational mandate 68(56.2%), and the least reported was isolation from peers 5 (4.1%).

## Discussion

Physiotherapists in this study held positive attitudes and beliefs about EBP as also indicated in previous studies by Salbach et al. (2007); Scurlock-Evans, Upton and Upton (2014); and Mota da Silva et al. (2015). This was observed despite differences in the practice settings, which varied across studies such as rehabilitation settings (Mota da Silva et al. 2015), acute care hospital setting (Salbach et al. 2007) and general hospital setting (as in our study). A higher educational qualification was positively associated with knowledge and skills of EBP in this study as also reported by Scurlock-Evans, Upton and Upton (2014). Contrary to the findings made by Jette et al. (2003), participants (PTs) of this study rated their confidence in research as very low, likewise critical appraisal of psychometrics and statistics. The differences in the self-efficacy ratings of PTs in relation to research and literature appraisal could be that most PTs in this study may not have acquired further knowledge in critical appraisal of research literature because continuous professional development programmes organised or attended in Ghana to date may not have fully addressed this topic. It is also possible that the lack of time and busy schedules at the various facilities may have caused this low confidence.

It is important to note that more than half of the participants stated that they have received formal training in critical appraisal of the literature; however, they reported low self-efficacy ratings in critically appraising the psychometric properties of outcome measures (20%), critically appraising the strengths and weaknesses of different study designs (19%) and interpreting results of statistical procedures such as t-tests and chi-square tests (18%) and linear or logistic regression (16%). The concern, according to the self-efficacy theory, is that PTs with low self-efficacy for searching and appraising research literature and integrating the findings into clinical practice are less likely to perform these activities than people who perceive their level of skill to be higher (Shea, Basch & Zybert 2008).

In this study, PTs who have practised for less than 5 years demonstrated better knowledge and skills of EBP compared with PTs with more than 8 years of practice. A study conducted by Manns et al. (2015) in Alberta, Canada, revealed similar results. A lower percentage of participants (PTs) perceived their organisation or facility to be supportive of using current research in practice; however, this support was not in the form of a mandate promoting research use. This outcome suggests that physiotherapy facilities or organisations in Ghana do not provide support for the implementation of EBP, especially in rural areas. The failure of facilities to make provision of such resources as stated by the participants in our study may be responsible for their low level in implementing EBP for stroke survivors. Bozzolan et al. (2014) reported similar results where the shortage of resources available in the workplace presented limitations in the implementation of EBP among health professionals in Italy. Overall, there is a need to develop a supportive organisational infrastructure in addition to enhancing skills of the evidence-based practitioner to increase research integration in physiotheraphy practice for stroke survivors (Rappolt et al. 2005).

This study’s findings highlighted potential practitioner and organisational barriers to the implementation of EBPT for stroke survivors with organisational barriers being the most reported. A notable practitioner-level barrier to the implementation of EBP was the lack of formal education and training in how to critically evaluate research literature. Without protected time or the skill to conduct literature searches, PTs cannot take advantage of Internet access to online databases, which were even less available to 83.5% of the PTs who participated in this study. A consistent observation was that PTs who work in community care centres, rural or non-teaching institutions are particularly disadvantaged regarding the implementation of EBP because of a lack of educational and human resources. Salbach et al. (2007) reported similar results in a study conducted in Canada.

Most of the PTs in this study stated, in descending order, the following as the five major barriers to implementing EBPT for stroke survivors: lack of an organisational mandate, insufficient time, lack of information resources, lack of understanding of statistics and lack of interest. In contrast, the most reported barrier to EBPT for stroke survivors according to some studies was insufficient time (Panhale & Bellare 2015; Scurlock-Evans et al. 2014).

There was a limited period for data collection. There is therefore the possibility that participants may have completed the questionnaire in haste in order to meet the time limit. Some participants who were contacted personally were reluctant to participate owing to the busy nature of their work and personal schedules.

## Conclusion

Lack of adequate resources, lack of organisational support and low self-efficacy to perform EBP activities represent barriers to implementing EBP for stroke survivors. This study found a positive relationship between educational qualification and knowledge and skills of EBP. In addition, the study showed that most physiotherapy facilities or organisations in Ghana were not supportive in the implementation of EBP for stroke survivors. These findings reinforce the need for continuing education to enhance skills and self-efficacy to search and critically evaluate the research literature among Ghanaian PTs. Overall, there is a need to develop a supportive organisational infrastructure to increase research integration in physiotherapist practice for stroke survivors in Ghana.

## Appendix 1: Practitioner and organisational barriers to evidence-based stroke rehabilitation questionnaire

1. Do you currently treat patients who have suffered a stroke?
  □ Yes     □ No

**If ‘No’, please return the questionnaire in the business-reply envelope enclosed so that we may remove you from our mailing list. You do not have to complete the questionnaire. Thank you.**

**This section inquires about your personal attitudes towards, use of, and perceived benefits and limitations of evidence-based practice (EBP). EBP is defined as ‘integrating individual clinical expertise with the best available external clinical evidence from systematic research’ (Sackett et al. 1996).**

For the following items, place a mark in the appropriate box that indicates your response.

2. Application of EBP is necessary in the practice of physical therapy.
  □ Strongly Disagree     □ Disagree     □ Neutral     □ Agree     □ Strongly Agree
3. Literature and research findings are useful in my day-to-day practice.
  □ Strongly Disagree     □ Disagree     □ Neutral     □ Agree     □ Strongly Agree
4. I need to increase the use of evidence in my daily practice.
  □ Strongly Disagree     □ Disagree     □ Neutral     □ Agree     □ Strongly Agree
5. The adoption of EBP places an unreasonable demand on physical therapists.
  □ Strongly Disagree     □ Disagree     □ Neutral     □ Agree     □ Strongly Agree
6. I am interested in learning or improving the skills necessary to incorporate EBP into my practice.
  □ Strongly Disagree     □ Disagree     □ Neutral     □ Agree     □ Strongly Agree
7. EBP improves the quality of patient care.
  □ Strongly Disagree     □ Disagree     □ Neutral     □ Agree     □ Strongly Agree
8. EBP helps me make decisions about patient care.
  □ Strongly Disagree     □ Disagree     □ Neutral     □ Agree     □ Strongly Agree
9. EBP does not take into account patient preferences (i.e. patients’ reported values and preferences for treatment).
  □ Strongly Disagree     □ Disagree     □ Neutral     □ Agree     □ Strongly Agree
10. Physiotherapists should be responsible for conducting their own literature reviews to answer their clinical questions.
  □ Strongly Disagree     □ Disagree     □ Neutral     □ Agree     □ Strongly Agree
11. Physiotherapists should be responsible for critically evaluating the quality of the literature to address their clinical questions.
  □ Strongly Disagree     □ Disagree     □ Neutral     □ Agree     □ Strongly Agree
12. Physiotherapists should be responsible for interpreting whether research findings apply to their individual patients.
  □ Strongly Disagree     □ Disagree     □ Neutral     □ Agree     □ Strongly Agree

**The following section inquires about your educational preparation and about how confident you are in your ability to access, interpret and apply research evidence to a clinical problem. The information you provide will be used to enhance curriculum development, plan continuing education courses and develop educational resources.**

For the following items, place a mark in the appropriate box that indicates your response.

13. I learned the foundations for EBP as part of my academic preparation.
  □ Strongly Disagree     □ Disagree     □ Neutral     □ Agree     □ Strongly Agree
14. I have received formal training (e.g. workshops, courses) in search strategies for finding research relevant to my practice.
  □ Strongly Disagree     □ Disagree     □ Neutral     □ Agree     □ Strongly Agree
15. I received formal training in how to critically evaluate research literature as part of my academic preparation.
  □ Strongly Disagree     □ Disagree     □ Neutral     □ Agree     □ Strongly Agree

For each of the following activities, please indicate how confident you are in your current level of ability by choosing the corresponding number on the following rating scale:

**Table:**

**0%**	**10%**	**20%**	**30%**	**40%**	**50%**	**60%**	**70%**	**80%**	**90%**	**100%**
**Cannot** **Do at All**					**Moderately** **Can Do**					**Certain** **Can Do**	

16. How confident are you in your ability to:
  1. … identify clinical problems following a patient assessment? ______%
  2. … formulate a question based on the clinical problem to guide a literature search? ______%
  3. … effectively search the relevant literature to address the question? ______%
  4. … critically appraise the literature for reliability and relevance? ______%
  5. … critically appraise the psychometric properties of outcome measures? ______%
  6. … critically appraise the strengths and weaknesses of different study designs? ______%
  7. … interpret results of statistical procedures such as *t* tests and chi-square tests? ______%
  8. … interpret results of statistical procedures such as linear or logistic regression? ______%
  9. … appropriately apply evidence from the literature to the individual patient? ______%
  10. … understand your patient’s needs and treatment preferences? ______%
  11. … decide on an appropriate course of action in collaboration with the patient? ______%
  12. … continually evaluate the effect of your practice? ______%

**The following section inquires about the availability of resources and support to promote EBP.**

For the following items, place a mark in the appropriate box that indicates your response.

17. I have access in my facility to current research through professional journals in their paper form.
  □ Yes     □ No     □ Do Not Know
18. I have the ability to access relevant databases and the Internet at my facility.
  □ Yes     □ No     □ Do Not Know
19. I have the ability to access relevant databases and the Internet at home or locations other than my facility.
  □ Yes     □ No     □ Do Not Know
20. A resource person (e.g. clinical practice leader, librarian, research therapist) is available at my facility to assist me with implementing EBP.
  □ Yes     □ No     □ Do Not Know
21. My facility mandates the use of current research findings in practice (mandate is a written requirement).
  □ Yes     □ No     □ Do Not Know
22. My facility provides protected time to conduct literature reviews and appraise the literature.
  □ Yes     □ No     □ Do Not Know
23. My facility provides financial support to attend educational meetings and conferences.
  □ Yes     □ No     □ Do Not Know
24. Colleagues within my department are sceptical of new EBPs.
  □ Strongly Disagree     □ Disagree     □ Neutral     □ Agree     □ Strongly Agree

**The following item inquires about the top 3 barriers to updating your clinical practice with new knowledge.**

25. Indicate the **three greatest barriers** to updating your clinical practice with new knowledge.
  □ Insufficient time provided by management
  □ Lack of information resources
  □ Lack of research skills
  □ Poor ability to critically appraise the literature
  □ Lack of generalisability of research findings to my patient population
  □ Inability to apply research findings to individual patients with unique characteristics
  □ Lack of understanding of statistical analyses
  □ Lack of support among my colleagues in my facility
  □ Lack of interest
  □ Lack of an organisational mandate
  □ Isolation from peers

**The following section inquires about personal demographic and practice information.**

26. How old are you?
  ______ years
27. What is your gender?
  □ Female     □ Male
28. What is your entry-level degree for physical therapy?
  □ Certificate     □ Bachelor’s     □ Entry-level master’s
  □ Other, please specify: ________________________________
29. In what year did you graduate? _______
30. What is your highest degree attained?
  □ Bachelor’s     □ Entry-level master’s     □ Applied or research master’s     □ Doctoral
  □ Other, please specify:_________________________________
31. For how many years have you been practising?
  _____ years
32. Do you supervise physical therapist students in your practice?
  □ Yes     □ No
33. In a typical week, how many hours do you work?
  □ < 20 h     □ 20–30 h     □ 31–40 h     □ > 40 h
34. In a typical day, how many patients do you see?
  □ < 5 patients     □ 5–10 patients     □ 11–15 patients     □ > 15 patients
35. In a typical day, approximately how many patients with stroke do you see?
  □ < 2 patients     □ 2–5 patients     □ 6–10 patients     □ > 10 patients
36. How many full-time physical therapists work in the facility in which you do the majority of your patient care?
  _____ physiotherapists
37. Which of the following **best** describes the location of the facility in which you perform the majority of your patient care?
  □ Rural (defined as > 30 miles or 40 km from a major city)
  □ Urban
  □ Suburban
38. Which of the following **best** describes the facility at which you do most of your patient care?
  □ General hospital
  □ Rehabilitation hospital/facility
  □ Long-term care facility
  □ Complex continuing care
  □ Community health centre
  □ Community care access centre
  □ Home visiting agency
  □ Private practice/clinic
  □ University/educational institution
  □ Consulting firm/agency
39. Is your setting a teaching institution (defined as an institution that provides student therapists with clinical rotations/training)?
  □ Yes     □ No
40. Do you work in a team that includes professionals from other disciplines?
  □ Yes     □ No
If ‘Yes’, is the team a stroke team (or neurorehabilitation team), specifically, a team that focuses primarily on the assessment and treatment of individuals with stroke?
  □ Yes     □ No

## References

[CIT0001] Bello A, 2011, ‘Utilizing research findings in physiotherapy: A call for gap bridging’, *Niger Post Graduate Medical Journal* 18, 54–58.

[CIT0002] Bozzolan M., Simoni G., Balboni M., Fiorini F., Bombardi S., Bertin N. et al., 2014, ‘Undergraduate physiotherapy students’ competencies, attitudes and perceptions after integrated educational pathways in evidence-based practice: A mixed methods study’, *Physiotherapy Theory Practice* 30, 557–571. 10.3109/09593985.2014.910285 24766584

[CIT0003] Burt R.K., Loh Y. & Cohen B, 2009, ‘Autologous non-myeloablative haemopoietic stem cell transplantation in relapsing-remitting multiple sclerosis: A phase I/II study’, *Lancet Neurology* 8, 244–260. 10.1016/S1474-4422(09)70017-1 19186105

[CIT0004] Dawes M., Summerskill W., Glasziou P., Cartabellotta A., Martin J., Hopayian K. et al., 2005, ‘Silicy statement on evidence-based practice’, *BMC Medical Education* 5(1), 12–21. 10.1186/1472-6920-5-1 15634359 PMC544887

[CIT0005] Flores-Mateo G. & Argimon J.M, 2007, ‘Evidence-based practice in postgraduate healthcare education: A systematic review’, *BMC Health Services research* 7, 119. 17655743 10.1186/1472-6963-7-119 PMC1995214

[CIT0006] Gorgon E.J., Barrozo H.G., Mariano L.G. & Rivera E.F, 2013, ‘Research evidence uptake in a developing country: A survey of attitudes, education and self-efficacy, engagement, and barriers among physical therapists in the Philippines’, *Journal Evaluation of Clinical Practice* 19, 789–790. 10.1111/j.1365-2753.2012.01849.x 22583741

[CIT0007] Iles R. & Davidson M, 2006, ‘Evidence-based practice: A survey of physiotherapists’ current practice’, *Physiotherapy Research International* 11, 93–103. 10.1002/pri.328 16808090

[CIT0008] Jette D.U., Bacon K., Batty C., Carlson M., Ferland A., Hemingway R.D. et al., 2003, ‘Evidence-based practice: Beliefs, attitudes, knowledge and behaviours of physical therapist’, *Physical Therapy* 83(9), 786–805. 12940766

[CIT0009] Kumar S., Grimmer-Somers K. & Hughes B, 2010, ‘The ethics of evidence implementation in health care’, *Physiotherapy Research International* 15, 96–102. 10.1002/pri.479 20564759

[CIT0010] Langhorne P., Bernhardt J. & Kwakkel G, 2011, ‘Stroke care 2: Stroke rehabilitation’, *The Lancet* 377, 1693–1702. 10.1016/S0140-6736(11)60325-5 21571152

[CIT0011] Manns P., Norton A.V. & Darrah J, 2015, ‘Cross-sectional study to examine evidence-based practice skills and behaviours of physical therapy graduates: Is there a knowledge-to-practice gap?’, *Physical Therapy* 5, 568–578. 10.2522/ptj.20130450 24810862

[CIT0012] McEvoy M.P., Williams M.T., Olds T.S., Lewis L.K. & Petkov J, 2011, ‘Evidence-based practice profiles of physiotherapists transitioning into the workforce: A study of two cohorts’, *BMC Medical Education* 11, 25–29. 10.1186/1472-6920-11-100 22126299 PMC3248363

[CIT0013] Mota da Silva T., Costa L., Garcia A. & Costa L, 2015, ‘What do physical therapists think about evidence-based practice? A systematic review’, *Manual Therapy* 20, 388–401. 10.1016/j.math.2014.10.009 25458142

[CIT0014] Panhale V.P. & Bellare B, 2015, ‘Evidence-based practice among physiotherapy practitioners in Mumbai, India’, *Education for Health* 28(2), 154–155. 26609020 10.4103/1357-6283.170119

[CIT0015] Pollock A., Campbell P., Baer G., Choo P., Fortser A. & Morris J, 2014, ‘Challenges in integrating international evidence relating to stroke rehabilitation: Experiences from a Cochrane systemic review’, *International journal of stroke* 9(8), 965–967. 10.1111/ijs.12339 25381686

[CIT0016] Rappolt S., Pearce K., McEwen S. & Polatajko H.J, 2005, ‘Exploring organizational characteristics associated with practice changes following a mentored online educational module’, *Journal of Continuing Education of Health Professionals* 25, 116–124. 10.1002/chp.16 16078810

[CIT0017] Sackett D.L., Rosenberg W.M., Gray J.A., Haynes R.B. & Richardson W.S, 1996, ‘Evidence based medicine: what it is and what it isn’t’, *BMJ*, 312(7023), 71–72. 8555924 10.1136/bmj.312.7023.71 PMC2349778

[CIT0018] Salbach N., Jaglal S., Korner-Bitensky N., Rappolt S. & Davis D, 2007, ‘Practitioner and organizational barriers to evidence-based practice of physical therapists for people with stroke’, *Journal of the American Physical Therapy Association* 87, 1284–1303. 10.2522/ptj.20070040 17684088

[CIT0019] Scurlock-Evans L., Upton P. & Upton D, 2014, ‘Evidence-based practice in physiotherapy: A systematic review of barriers, enablers and interventions’, *Physiotherapy* 100, 208–219. 10.1016/j.physio.2014.03.001 24780633

[CIT0020] Shea S., Basch C.E. & Zybert P, 2008, ‘Correlates of internists’ practices in caring for patients with elevated serum cholesterol’, *American Journal of Health Promotion* 4, 421–428. 10.4278/0890-1171-4.6.421 22204620

[CIT0021] Silva T.M., Costa L.C. & Costa L.O, 2015, ‘Evidence-based practice: A survey regarding behaviour, knowledge, skills, resources, opinions and perceived barriers of Brazilian physical therapists from São Paulo state’, *Brazilian Journal of Physical Therapy* 19, 294–303. 10.1590/bjpt-rbf.2014.0102 26443977 PMC4620978

[CIT0022] Teasell R., Foley N., Salter K., Richardson M., Allen L., Hussein N. et al., 2015, ‘Evidence based review of stroke rehabilitation’, *Physiotherapy* 12, 1–35.

[CIT0023] Wilkinson I.A., Burridge J., Strike P. & Taylor P, 2015, ‘A randomised controlled trial of integrated electrical stimulation and physiotherapy to improve mobility for people less than 6 months post stroke’, *Disability and Rehabilitation* 10(6), 468–474. 10.3109/17483107.2014.917125 24827386

